# A hybrid approach based on deep learning and level set formulation for liver segmentation in CT images

**DOI:** 10.1002/acm2.13482

**Published:** 2021-12-06

**Authors:** Zhaoxuan Gong, Cui Guo, Wei Guo, Dazhe Zhao, Wenjun Tan, Wei Zhou, Guodong Zhang

**Affiliations:** ^1^ School of Computer Shenyang Aerospace University Shenyang China; ^2^ Key Laboratory of Intelligent Computing in Medical Image Ministry of Education Northeastern University Shenyang China

**Keywords:** active contour model, convolutional neural networks, CT image, fractional differential, liver segmentation

## Abstract

Accurate liver segmentation is essential for radiation therapy planning of hepatocellular carcinoma and absorbed dose calculation. However, liver segmentation is a challenging task due to the anatomical variability in both shape and size and the low contrast between liver and its surrounding organs. Thus we propose a convolutional neural network (CNN) for automated liver segmentation. In our method, fractional differential enhancement is firstly applied for preprocessing. Subsequently, an initial liver segmentation is obtained by using a CNN. Finally, accurate liver segmentation is achieved by the evolution of an active contour model. Experimental results show that the proposed method outperforms existing methods. One hundred fifty CT scans are evaluated for the experiment. For liver segmentation, Dice of 95.8%, true positive rate of 95.1%, positive predictive value of 93.2%, and volume difference of 7% are calculated. In addition, the values of these evaluation measures show that the proposed method is able to provide a precise and robust segmentation estimate, which can also assist the manual liver segmentation task.

## 1 INTRODUCTION

The accurate segmentation of liver is important not only for radiation therapy planning but also for follow‐up evaluations.^1^ Liver segmentation from CT volumes is difficult because the intensity contrast between liver and its surrounding tissues is obscure.^2^ Quantification research in structural neuroimaging can benefit from accurate liver segmentation of human abdomen CT images, which is also vital to the success of computer‐aided surgeries.

Recently, several liver segmentation methods have been proposed. Li et al.^3^ proposed an intensity bias and position constraint‐based level set model for liver segmentation. The level set model was used for initial liver segmentation. Graph cut was then applied to further optimize the segmentation results. Rafiei et al.^4^ combined 3D region growing and contrast enhancement algorithm to segment liver region. Tang et al.^5^ designed a multi‐scale CNN model for liver segmentation. The experimental results showed that their method was an effective way for liver segmentation. Peng et al.^6^ used graph cuts and a multi‐region‐based approach to obtain the liver surface. The segmentation was achieved by using an energy function which incorporates both region information and boundary. Mostafa et al.^7^ proposed an artificial bee colony optimization algorithm for liver segmentation. The centroids of clusters in the image were calculated by the artificial bee colony method. Mathematical morphology and region growing were then applied to achieve the final segmentation. Yan et al.^8^ used single statistical atlas registration to obtain an initial liver segmentation. Chemical shift‐based method was then applied for final segmentation. Wang et al.^9^ develop a priori statistical shape model for liver segmentation. The boundary information, the intensity information, and the sparse information were constructed to accurately segment the liver region. Ali et al.^10^ utilized artificial bee colony model and grey wolf optimization model for liver segmentation. The experiments showed that their method can obtain good results when applied to segment medical images. Goceri^11^ proposed a variational level set‐based model for liver segmentation. An adaptive‐signed pressure force function and a Sobolev gradient‐based model were jointly used for level set evolution. The experiment results showed that the level set contour can shrink to the edge of the liver accurately. Abd‐Elaziz et al.^12^ designed a region‐growing‐based method for liver segmentation. In their method, intensity analysis and preprocessing steps were combined to obtain the liver region. Yuan et al.^13^ proposed a fast marching and improved fuzzy cluster method for liver segmentation. Fast marching method and convex hull algorithm were used for initial liver's boundary detection. An improved fuzzy cluster method was then applied for refine the segmentation result. Wang et al.^14^ presented a sparse dictionary and hole filling method for liver segmentation. Sparse coding was used to obtain the initial liver boundary of the image, and a hole filling method was designed for liver boundary completion and smoothing to obtain the final segmentation results. Mir et al.^15^ proposed an automatic liver segmentation model. In their method, adaptive filter was used to reduce noise. Three dimensional region growing and the combination of morphological operators were combined to obtain the liver region. Chartrand et al.^16^ presented a laplacian mesh optimization method for liver segmentation. The initial liver contour was obtained by manual delineation. Laplacian mesh optimization was then used to refine the segmentation. Zareei and Karimi^17^ used a preprocessing model to obtain an initial segmentation close to the liver's boundary and then implemented a combination of gradient vector flow and balloon energy to improve the initial segmentation. Kitrungrotsakul et al.^18^ proposed a graph model for liver segmentation. Clustering algorithm was applied to construct graph which can further reduce the computational time. And liver segmentation can be achieved by their graph cut model. Altarawneh et al.^19^ proposed an improved distance regularization level set model for liver segmentation. In their method, a new balloon force was designed to discourage the evolving contour from exceeding the liver boundary, which can improve the segmentation accuracy effectively. Qin et al.^20^ proposed an intensity‐based CNN for liver segmentation. An entropy‐based saliency map was built by multinomial classification, and CNN was constructed and trained to predict the probability map of the liver boundary. Silva et al.^21^ used linear iterative clustering algorithm and probabilistic atlas in a deep convolutional neural networks (CNNs) to obtain an initial liver contour; 3D Chan‐Vese active contour model was then applied to acquire the final segmentation. Feng et al.^22^ used simple U‐net model for liver segmentation, and the experiment results showed the effectiveness of their method. Gloger et al.^23^ presented a fully automatized method for liver segmentation, which combined model knowledge and probability maps to delineate the liver contour. Ali et al.^24^ proposed a clustering and energy optimization model for liver segmentation. The experiment results demonstrated that their method obtained better mean values in terms of Jaccard Index and Dice Coefficient. Mostafa et al.^25^ proposed a whale optimization algorithm for liver segmentation. Whale optimization algorithm can remove a great part of non‐liver region from the image. Liver region was extracted by user interaction, and the morphological operations refined the final segmentation. Saito et al.^26^ developed a statistical shape model for liver segmentation. The statistical shape model‐guided expectation‐maximization algorithm was first used to obtain the initial liver boundary; graph cut was then applied to refine the segmentation. Eapen et al.^27^ proposed a Bayesian level set framework for liver segmentation. The level set contour was initialized by Bayesian probability model, level set evolution was achieved by using an energy function. Zheng et al.^28^ proposed a texture feature‐based method to extract the liver region; the liver boundary was obtained by the random walk algorithm. In the work by Yang et al.^29^ the value information and the spatial relationship between pixels were utilized to extract the liver region. A parallel algorithm was designed for further refining the segmentation. Trabelsi et al.^30^ proposed an active shape model to obtain the liver region. B‐spline registration was first applied to obtain the initial liver region. Active shape model was then applied to obtain the accurate liver segmentation. Although previous works have made great progress in improving the segmentation accuracy, most of them fail to extract the boundary of the liver accurately. In our method, an intensity constrained level set model is designed to refine the segmentation of the output of the CNN. The level set contour can be close to the liver boundary during the evolution, which increases the segmentation accuracy effectively.

**FIGURE 1:**
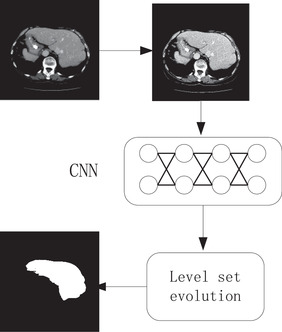
The pipeline of the proposed framework

In this paper, we propose to develop a fully automatic method for liver segmentation. First, fractional differential is used to enhance the image. A deep CNN is then applied to extract the initial liver region. Maximum connectivity model is designed to refine the segmentation. The final segmentation is achieved by the level set evolution. Figure 1 shows the pipeline of the proposed framework Figures 1.

**FIGURE 2:**
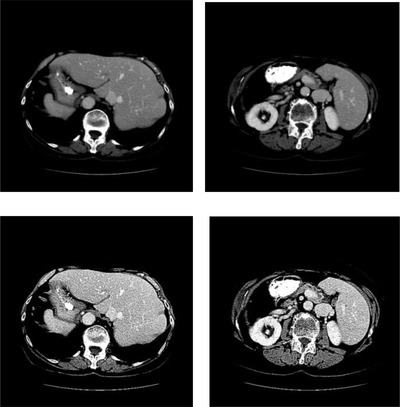
Examples of fractional differential enhancement. The first row: original images; second row: results after applying fractional differential enhancement

**FIGURE 3:**
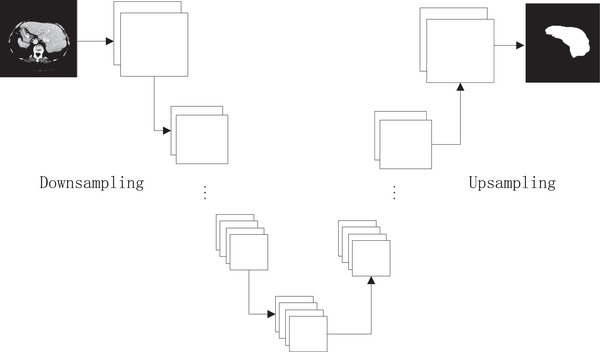
Structure of the convolutional neural network

## 2 MATERIALS AND METHODS

### 2.1 Fractional differential enhancement

Fractional differential is used as preprocessing step so that the contrast of liver and other tissues can be enhanced in each transaxial slice. Let ζ(t) be a signal, *t* is the discrete variable, *t* = 1, 2,…n, and the differential operator *v* can be denoted by:

(1) $$\begin{array}{ccc} \frac{d^{v}\zeta \left(t\right)}{dt^{v}} & \approx & \zeta \left(t\right)+\left(-v\right)\zeta \left(t-1\right)+\frac{-v(1-v)}{2}\zeta \left(t-2\right)\\ & & +\;\frac{-v(1-v)(2-v)}{6}\zeta \left(t-3\right)+\cdots \end{array}$$

In the area of digital image, fractional differential can be defined as:

(2) $$\begin{array}{ccc} \frac{d^{v}\zeta \left(x,y\right)}{dx^{v}} & \approx & \zeta (x,y)+(-v)\zeta (x-1,y)\\ & & +\;\frac{-v(1-v)}{2}\zeta \left(x-2,y\right)+\frac{-v(1-v)(2-v)}{6}\\ & & \times \;\zeta (x-3,y)+\cdots \end{array}$$

(3) $$\begin{array}{ccc} \frac{d^{v}\zeta \left(x,y\right)}{dy^{v}} & \approx & \zeta (x,y)+(-v)\zeta (x,y-1)\\ & & +\;\frac{-v(1-v)}{2}\zeta \left(x,y-2\right)+\frac{-v(1-v)(2-v)}{6}\\ & & \times \;\zeta (x,y-3)+\cdots \end{array}$$

The fractional differential operator is constructed to preserve the low‐frequency contour features of the liver region and improve the overall texture. Given an image ζ(x,y), the fractional differential enhancement image ψ(x,y) in our method is designed as:

(4) $$\begin{array}{ccc} \psi (x,y) & = & 8\zeta (x,y)-v(\zeta (x-1,y-1)+\zeta (x,y-1)\\ & & +\;\zeta (x+1,y-1)+\zeta (x-1,y)\zeta (x+1,y)\\ & & +\;\zeta (x-1,y+1)+\zeta (x,y+1)+\zeta (x+1,y+1))\\ & & +\;\frac{v^{2}-v}{2}(\zeta \left(x-2,y-2\right)+\zeta \left(x-2,y\right)\\ & & +\;\zeta (x-2,y+2)+\zeta (x,y-2)+\zeta (x,y+2)\\ & & +\;\zeta (x+2,y-2)+\zeta (x+2,y)+\zeta (x+2,y+2)) \end{array}$$

where v is the order differentiation operator. Fractional differential enhancement highlights the fine details of the object, which can improve the contrast between liver and the surrounding tissues.^31^ Fig. 2, 3 exhibits the result of fractional differential enhancement.

### 2.2 Convolutional neural networks

The proposed CNN model is an 11‐layer deep structure, which is composed of down‐sampling stage and up‐sampling stage. The down‐sampling stage adopts several convolutional layers, each followed by a rectified linear unit (ReLU), and the kernels of max‐pooling is 2 × 2. After training the network, the connected component analysis is used to divide all labeled voxels into several connected components; the largest component is selected as the final liver region. We fine tune the network with the following parameters: batch size = 2, base learning rate = 0.00001, epoch = 10, Adam, and Relu are used as the optimizer and the activation function, respectively.

### 2.3 Level set evolution

Distance regularized level set evolution intensity constrained (DRLSE)^32^ is used in our level set model. Based on the DRLSE model, we designed an intensity‐constrained term which can guide the evolution of the level set contour. The final liver segmentation can be achieved by the evolution of DRLSEIC model. An edge‐based information is used to define the external energy.

Let U be an image on a domain Ω, we define an edge indicator function g by

(5) $$g=\frac{1}{1+{\left|\nabla G_{\sigma }*U\right|}^{2}}$$

where $G_{\sigma }$ is a Gaussian kernel with a standard deviation σ.

The energy functional of DRLSE model is defined as follows:

(6) E(ϕ)=αP(ϕ)+λL(ϕ)+βA(ϕ)

Where α,λ, and β are positive parameters and fixed in this study.

The energy functional L(ϕ),A(ϕ), and P(ϕ) are defined by:

(7) $$L\left(\phi \right)=\int_{\Omega }g\delta \left(\phi \right)\left|\nabla \phi \right|dx$$

(8) $$A\left(\phi \right)=\int_{\Omega }gH(-\phi )dx$$

(9) $$P\left(\phi \right)=\int_{\Omega }p(\left|\nabla \phi \right|)dx$$

where δ and H are the Dirac delta function and the Heaviside function, respectively, *p* is a potential function:$p\left(s\right)=s^{2}$. P(ϕ),L(ϕ),A(ϕ) are the penalty term, the length term, and the area term, respectively.

The regularized versions of H(·) and δ(·) are defined as:

(10) $$\left\{\begin{array}{c} H_{\varepsilon }\left(x\right)=\frac{1}{2}\left(1+\frac{2}{\pi }\arctan\left(\frac{x}{\varepsilon }\right)\right)\\ \delta_{\varepsilon }\left(x\right)=\frac{1}{\pi }\frac{\varepsilon }{x^{2}+\varepsilon^{2}} \end{array}\right.$$

The parameter ε is usually set to 1.5.

The output of CNN can be viewed as a label image Y, which is a binary map such that Y(κ)=1 for κ in the label region and Y(κ)=0 otherwise. For a label image Y, we let the level set function ϕ take negative values for κ∈{κ:Y(κ)=1}, and positive values for κ∈{κ:Y(κ)=0}. Therefore, the zero level contour of the level set function ϕ can be viewed as the boundary of the region of interest (ROI), which is labeled by Y. The zero level contour is denoted by C.

The initial liver class can be obtained by the statistical information of image Y, which is defined as:

(11) $$S=N(\mu_{\mathrm{liver}},\sigma_{\mathrm{liver}}^{2})$$

where $\mu_{\mathrm{liver}}$ is the mean intensity value of the liver class, and $\sigma_{\mathrm{liver}}$ is its variance.

Then, the intensity range of the liver region can be estimated by:

(12) $$Y_{\mathrm{low}}=\mu_{\mathrm{liver}}-w_{1}\times \sigma_{\mathrm{liver}}$$

(13) $$Y_{\mathrm{high}}=\mu_{\mathrm{liver}}+w_{2}\times \sigma_{\mathrm{liver}}$$

An intensity constrained term is designed based on the intensity range of the liver region. The energy of the intensity‐constrained term is designed as:

(14) $$\chi \left(\phi \right)=\eta \int_{\Omega }\left[\frac{1+\Delta (Y(x))}{2}-\frac{1-\Delta (Y(x))}{2}\right]H\left(\phi \right)dx$$

(15) $$\Delta \left(x\right)=\left\{\begin{array}{c} 1,\;x\in (Y_{\mathrm{low}},Y_{\mathrm{high}})\\ -1,\;\;\;else\; \end{array}\right.$$

The intensity‐constrained term enables the level set contour to evolve inside the liver region, which can improve the segmentation accuracy effectively.

The final energy function of DRLSEIC model is formulated as follows:

(16) $$\begin{array}{ccc} F(\phi ) & = & E\left(\phi \right)+X\left(\phi \right)=\lambda \int_{\Omega }g\delta (\phi )\left|\nabla \phi \right|dx\\ & & +\;\beta \int_{\Omega }gH(-\phi )dx+\alpha \int_{\Omega }p(\left|\nabla \phi \right|)dx\\ & & +\;\eta \int_{\Omega }\left[\frac{1+\Delta (Y(x))}{2}-\frac{1-\Delta (Y(x))}{2}\right]H\left(\phi \right)dx \end{array}$$

This energy functional (16) can be minimized by solving the following gradient flow:

(17) $$\begin{array}{ccc} \frac{\partial \phi }{\partial t} & = & \alpha div(d_{p}\left(|\nabla \phi |)\nabla \phi \right)+\lambda \delta_{\varepsilon }\left(\phi \right)div\left(g\frac{\nabla \phi }{\left|\nabla \phi \right|}\right)\\ & & +\;\beta g\delta_{\varepsilon }\left(\phi \right)+\eta \left[\frac{1+\Delta (Y(x))}{2}-\frac{1-\Delta (Y(x))}{2}\right]\delta_{\varepsilon }\left(\phi \right) \end{array}$$

## 3 RESULTS

Our method has been validated on two databases 3D‐IRCADband LiTs 2017. The LiTS dataset provides 130 scans and segmentation labels for liver. And 3D‐IRCADb dataset provides 20 scans. One hundred ten subsets were used for training, and 40 subsets were used for testing. The training data and the testing data were separated. Segmented tumor and liver are merged into the whole liver. The data were collected from different hospitals, and the resolution of the CT scans varies between 0.45 mm and 6 mm for intra‐slice and between 0.6 and 1.0 mm for inter‐slices (512 × 512pixels), respectively.^2^ Unless otherwise specified, the following parameters are fixed in this paper: $v=0.6,\alpha =1,\lambda =1,\beta =1,\varepsilon =1,w_{1}=1,w_{2}=1.2,\eta =3$, The computation was done on a Windows 10 server with an Intel Xeon silver 4210R CPU (2.4 GHz and 64 GB memory) and Nvidia GPU GeForce Titian RTX.

### 3.1 Effectiveness of the proposed method

**FIGURE 4:**
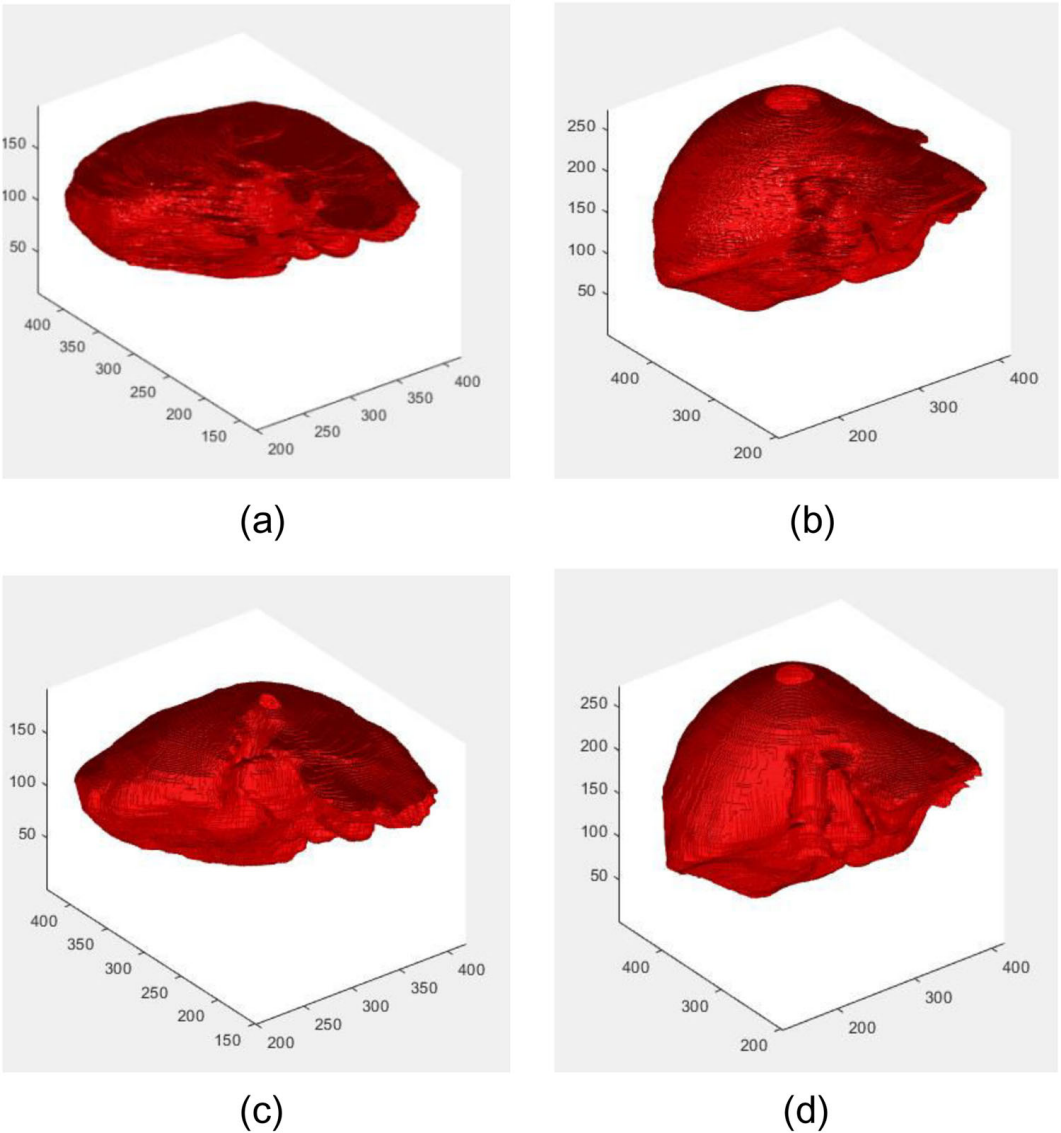
3D view of the segmentation results for liver labels of three test images using our method. (a and b) The segmentation results by our method. (c and d) The corresponding manual segmentation

**FIGURE 5:**
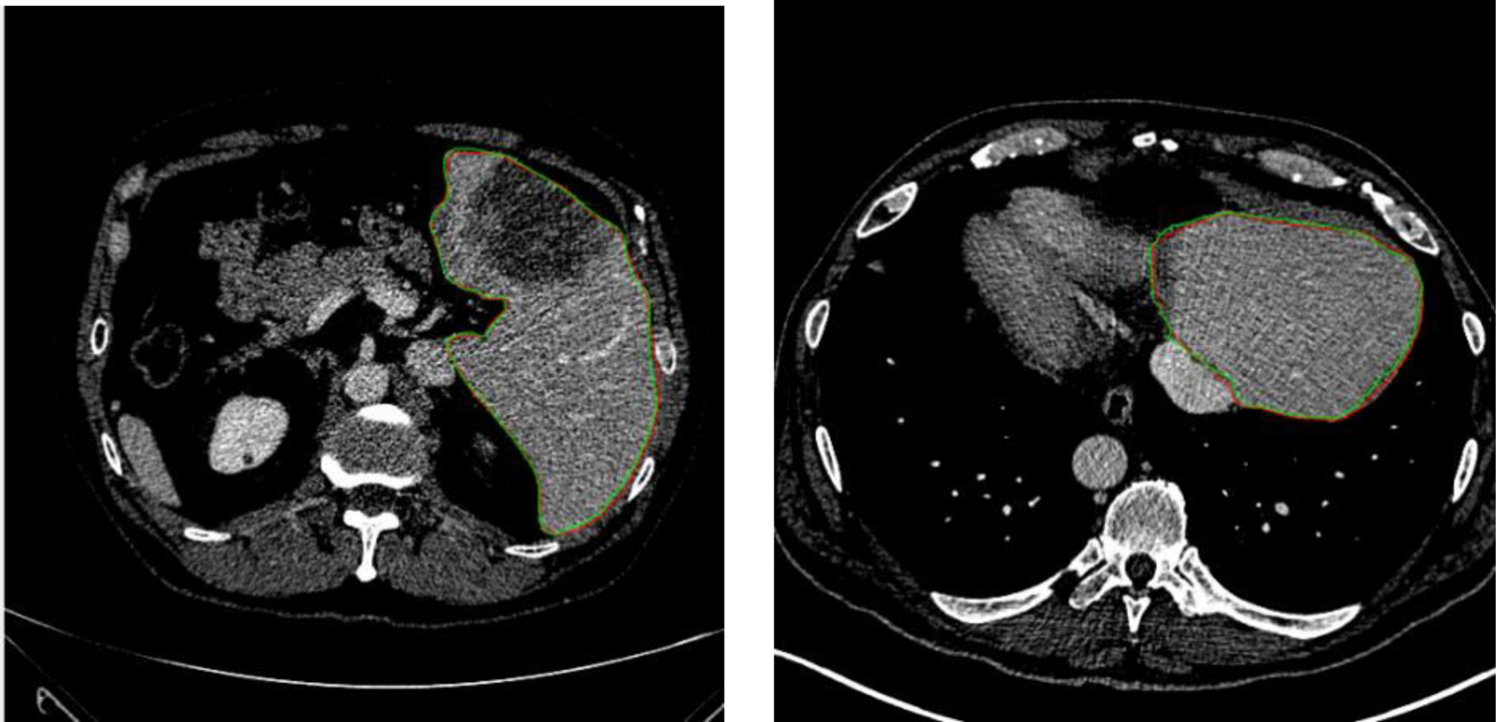
Coronal view of the segmentation results of liver labels by our method

Figure 4 shows three liver labels segmentation results of the proposed method. Figure 4a,b is the segmentation results obtained by our method. Figure 4c,d is the corresponding manual segmentations. It can be seen that the results of our method are quite similar to those of the manual segmentations. Figure 5 exhibits the coronal view of segmentation results for the liver of one test image using our method. The Green lines and the red lines are the manual segmentation and the proposed method's segmentation, respectively. From the picture we can see that the proposed method's segmentation is very close to the manual segmentation.

**FIGURE 6:**
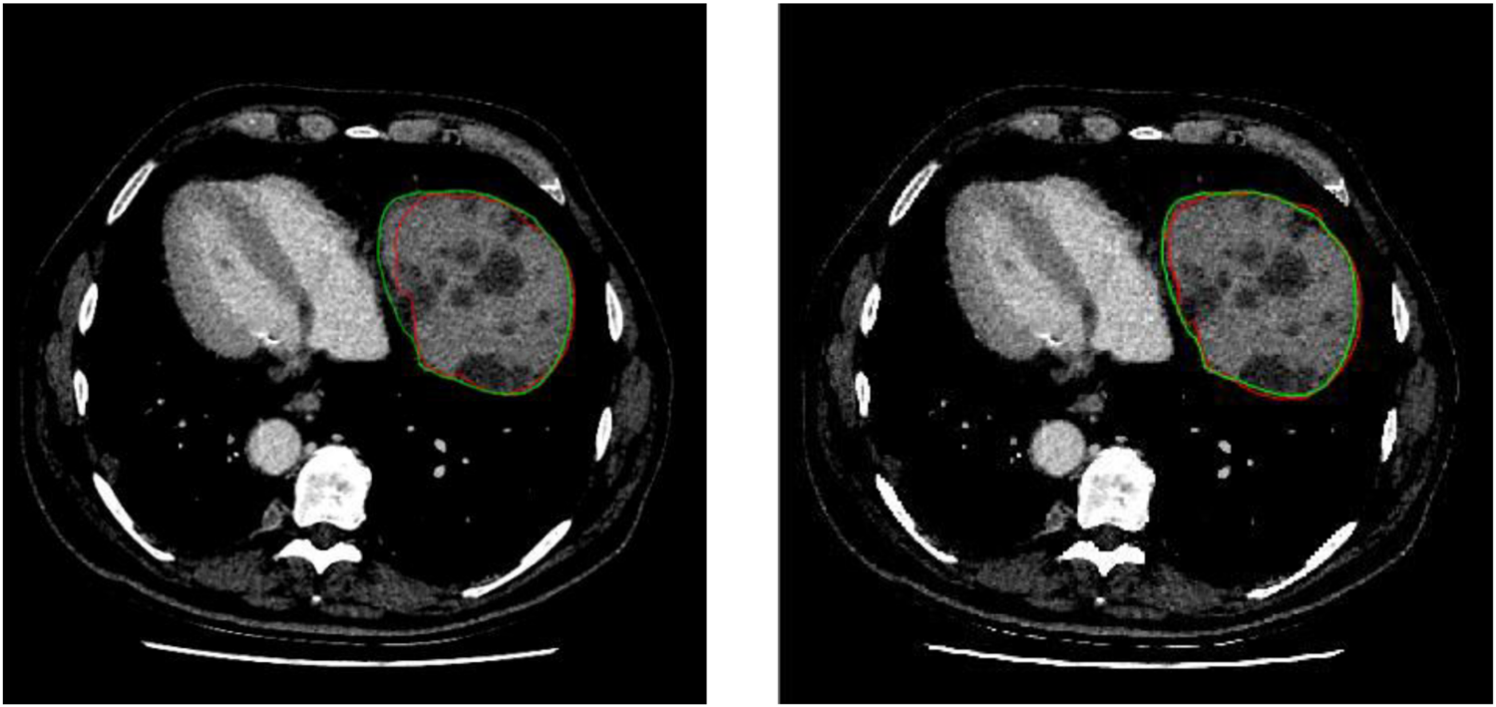
Example of a liver segmentation using our method. (a) Results of convolutional neural network (CNN); (b) results of CNN + DRLSEIC

We compared the performance of CNN + DRLSE with CNN on the same training and testing sets. An example of the segmented liver in a subject is illustrated in Figure 6. It can be seen that CNN model (Figure 6a, red line) produces poor segmentations on certain areas, mainly because of the low contrast between those areas and other segmented region. The result of CNN + DRLSEIC (Figure 6b, red line) is mostly overlapping with the ground‐truth segmentation (green line) and shows fewer false‐positive labeling.

### 3.2 Qualitative evaluation of the segmentation accuracy

Five image spatial metrics were adopted to evaluate the algorithm performance between automatic and manual segmentation,^33^ namely Dice Coefficient (DC), true positive rate (TPR), volume difference (VD), Jacard Index (JI), and positive predictive value rate (PPV). The definitions of each of the image metrics are given in Equations (18), (19), (20), (21), and 22, respectively.

(18) $$\mathrm{DC}=\frac{2\left|S\cap G\right|}{\left|S\right|+\left|G\right|}$$

(19) $$\mathrm{TPR}=\frac{\left|S\cap G\right|}{G}$$

(20) $$\mathrm{VD}=\frac{\left|S-G\right|}{G}$$

(21) $$\mathrm{PPV}=\frac{S\cap G}{S\cap G+S\cap \bar{G}}$$

(22) $$\mathrm{JI}=\frac{\left|S\cap G\right|}{\left|S\cup G\right|}$$

where *S* is the segmentation result, *G* is the ground truth, and $\bar{G}$ is the complement operator of G.

The border voxels of the segmentation and the ground truth are represented as $S_{\mathrm{seg}}$, $S_{\mathrm{truth}}$. For each voxel *p* along a given border, the closest voxel along the corresponding border in the other result is given by $D_{\min}\left(p,S_{\mathrm{truth}}\right)$, $p\in S_{\mathrm{seg}}$ or $D_{\min}\left(p,S_{\mathrm{seg}}\right)$, $p\in S_{\mathrm{truth}}$.

The mean surface distance is defined as:

(23) $$\begin{array}{ccc} & & \mathrm{MSD}(S_{\mathrm{seg}},S_{\mathrm{truth}})\\ & & \;=\frac{\sum_{p\in S_{\mathrm{seg}}}D_{\min}\left(p,S_{\mathrm{truth}}\right)+\sum_{p\in S_{\mathrm{truth}}}D_{\min}\left(p,S_{\mathrm{seg}}\right)}{N_{1}+N_{2}}\;\; \end{array}$$

where N1 and N2 are the numbers of voxels on the border surfaces of the segmentation and ground truth.

The hausdorff surface distance (HSD) is similar to the mean surface distance (MSD), which is defined as:

(24) $$\begin{array}{ccc} & & \mathrm{HSD}(S_{\mathrm{seg}},S_{\mathrm{truth}})\\ & & \;=\max[D_{\min}\left(S_{\mathrm{truth}},S_{\mathrm{seg}}\right),D_{\min}\left(S_{\mathrm{seg}},S_{\mathrm{truth}}\right)]\; \end{array}$$

**FIGURE 7:**
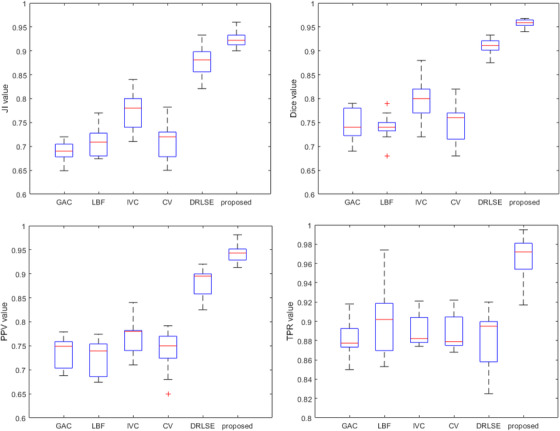
Quantitative comparison of the proposed method with CV, LBF, distance regularized level set evolution (DRLSE), IVC, and GAC

The performance of our method was compared against five state‐of‐the‐art methods: chan‐vese (CV) model,^34^ geodesic active contours (GAC) model,^35^ DRLSE^36^ model, selective binary and gaussian filtering regularized level set (SBGFRLS)^37^ model, and local binary fitting (LBF)^38^ model. It can be seen from Figure 7 that our proposed approach yielded average Dice, JI, PPV, and TPR, respectively. The median dice scores reach 0.961 for the proposed method, followed by 0.912 for DRLSE, 0.763 for CV model, 0.772 for image visual control (IVC) model, 0.744 for LBF model, and 0.752 for GAC model. The median JI scores reach 0.941 for the proposed method, followed by 0.884 for DRLSE, 0.733 for CV model, 0.779 for IVC model, 0.714 for LBF model, and 0.682 for GAC model. The median PPV scores reach 0.948 for the proposed method, followed by 0.894 for DRLSE, 0.748 for CV model, 0.77 for IVC model, 0.742 for LBF model, and 0.751 for GAC model. The median TPR scores reach 0.978 for the proposed method, followed by 0.891 for DRLSE, 0.879 for CV model, 0.883 for IVC model, 0.914 for LBF model, and 0.878 for GAC model. All the five state‐of‐the‐art methods produced non‐liver region during level set evolution; the proposed method can control the level set contour to evolve inside the liver region. Therefore, the proposed method outperformed other methods in terms of the above several metrics.

The VD values of liver segmentation are presented in Table 1. It can be seen that the proposed method obtained a very low VD value for most of the cases. However, it is obvious that case 05 and case 27 received unsatisfactory results, mainly because more misclassified voxels were produced, which led to a significant decrease in the quantity of the VD values.

**TABLE 1:** The detail index of the proposed method and manual segmentation in terms of volume difference

Dataset	VD (%)	Dataset	VD (%)	Dataset	VD (%)	Dataset	VD (%)
Case 01	0.055	Case 11	0.04	Case 21	0.028	Case 31	0.047
Case 02	0.027	Case 12	0.037	Case 22	0.068	Case 32	0.015
Case 03	0.011	Case 13	0.006	Case 23	0.046	Case 33	0.022
Case 04	0.083	Case 14	0.048	Case 24	0.041	Case 34	0.023
Case 05	0.112	Case 15	0.137	Case 25	0.081	Case 35	0.061
Case 06	0.072	Case 16	0.022	Case 26	0.077	Case 36	0.039
Case 07	0.045	Case 17	0.013	Case 27	0.194	Case 37	0.019
Case 08	0.077	Case 18	0.017	Case 28	0.052	Case 38	0.017
Case 09	0.092	Case 19	0.058	Case 29	0.036	Case 39	0.051
Case 10	0.053	Case 20	0.034	Case 30	0.044	Case 40	0.083

Abbreviation: VD, volume difference.

The number of convolutional layer and up‐sampling layer had great impact on the segmentation accuracy of a CNN. To select an optimal structure, four different convolutional layer and up‐sampling layer were validated. Resulting evaluation metrics are summarized in Table 2. From the table, we can observe that the structure of 5 conv&5 up‐sampling receives best performance. The input image size is 512×512, when 6 max pooling are applied, it is difficult to extract features from the feature map when 6 max pooling are applied. Therefore, the performance of the proposed CNN reduced with more extent compared with using five layers structure.

**TABLE 2:** Accuracy for different numbers of convolutional layers and up‐sampling layers

Metrics	3 conv&3 up‐sampling	4 conv&4 up‐sampling	5 conv&5 up‐sampling	6 conv&6 up‐sampling
Dice (%)	0.90 ± 0.03	0.91 ± 0.02	0.958 ± 0.021	0.84 ± 0.05
TPR (%)	0.87 ± 0.03	0.835 ± 0.04	0.971 ± 0.022	0.911 ± 0.042
VD (%)	0.15 ± 0.03	0.15 ± 0.05	0.05 ± 0.034	0.35 ± 0.06
JI (%)	0.82 ± 0.02	0.835 ± 0.02	0.921 ± 0.021	0.721 ± 0.061
PPV (%)	0.961 ± 0.03	0.955 ± 0.04	0.952 ± 0.031	0.912 ± 0.021
MSD (mm)	15.33 ± 4.13	11.91 ± 2.27	9.58 ± 2.97	12.77 ± 3.35
HSD (mm)	5.74 ± 0.92	4.94 ± 1.32	3.44 ± 1.09	5.04 ± 1.03

Abbreviations: JI, Jacard Index; PPV, positive predictive value; TPR, true positive rate; VD, volume difference.

The results of different network structure in terms of several evaluation metrics are recorded in Table 2. The comparison of the values of these metrics shows that the network structure of using five convolutional layers and five up‐sampling layers gave more robust performance, achieving a mean Dice of 0.958±0.021, a mean TPR of 0.971±0.022, a mean VD of 0.05±0.034, a mean JI of 0.921±0.021, and a mean PPV of 0.952±0.031. Based on this experiment, a network of five convolutional layers and five up‐sampling layers was established as the optimal structure of the proposed CNN.

We exhibit the influence of the level set model on segmentation accuracy in Table 3 and present the comparison of dice values with and without the level set model. It can be observed that the level set model can increase the segmentation accuracy by 1–2 percent. The reason lies in that the proposed level set model can detect clearer boundaries and thus improve the segmentation results.

**TABLE 3:** Comparison of our model with and without the level set evolution

Metrics	CNN	CNN + DRLSEIC
Dice (%)	0.941 ± 0.014	0.952 ± 0.017
TPR (%)	0.933 ± 0.021	0.944 ± 0.015
VD (%)	0.14 ± 0.03	0.09 ± 0.015
JI (%)	0.872 ± 0.011	0.891 ± 0.021
PPV (%)	0.914 ± 0.015	0.942 ± 0.019
MSD (mm)	11.12 ± 3.04	9.52 ± 2.74
HSD (mm)	4.28 ± 1.02	3.28 ± 0.92

Abbreviations: CNN, convolutional neural network; JI, Jacard Index; PPV, positive predictive value; TPR, true positive rate; VD, volume difference.

We compared our method with other four CNN models. Table 4 shows results for the U‐net, U‐net++, Segnet, fully convolutional networks (FCN), and the proposed method. For a fairly comparison, we used five convolution layers for each model. The size of kernel was 3. From the table, we can see that the proposed network offered the most accurate segmentation results in comparison to the other four CNN methods in terms of Dice, TPR,VD, JI, and PPV.

**TABLE 4:** Comparison of different CNN segmentation methods

Metrics	U‐net	U‐net++	Segnet	FCN	Proposed
Dice (%)	0.91 ± 0.03	0.931 ± 0.03	0.901 ± 0.02	0.82 ± 0.05	0.958 ± 0.02
TPR (%)	0.88 ± 0.03	0.941 ± 0.03	0.931 ± 0.02	0.891 ± 0.03	0.951 ± 0.02
VD (%)	0.12 ± 0.03	0.07 ± 0.04	0.15 ± 0.04	0.38 ± 0.05	0.07 ± 0.02
JI (%)	0.85 ± 0.02	0.875 ± 0.03	0.781 ± 0.02	0.691 ± 0.03	0.901 ± 0.03
PPV (%)	0.961 ± 0.03	0.955 ± 0.04	0.912 ± 0.02	0.902 ± 0.04	0.931 ± 0.02
MSD (mm)	12.33 ± 2.83	10.08 ± 3.02	13.48 ± 3.56	15.77 ± 4.65	9.27 ± 3.38
HSD (mm)	4.48 ± 1.12	3.94 ± 1.02	4.74 ± 1.19	5.04 ± 1.03	3.13 ± 0.98

Abbreviations: CNN, convolutional neural network; JI, Jacard Index; PPV, positive predictive value; TPR, true positive rate; VD, volume difference.

In our paired *t*‐tests, the significance level was set as 0.05. The *p*‐values for the paired *t*‐tests are summarized in Table 5. The *p*‐values of paired *t*‐tests show that the difference between our proposed method and the other three methods is significant.

**TABLE 5:** *p*‐values of paired *t*‐tests between our model and other four methods for Dice values

Metrics	Dice
U‐net vs. Ours	$10^{-3}$
U‐net++ vs. Ours	$10^{-2}$
Segnet vs. Ours	$10^{-3}$
FCN vs. Ours	$10^{-4}$

## 4 DISCUSSION

The novel hybrid semi‐automatic method proposed in the present study showed high accuracy in liver extraction. However, the evolution of the level set model is time‐consuming. In the future, we will try to accelerate the level set evolution with Compute Unified Device Architecture. Based on our liver segmentation results, we can identify tumor and vessels from the liver region. The proposed model can be implemented in a preoperative virtual liver surgery planning system to assist a surgeon to make an optimal treatment plan for a patient. The proposed method does not require any preprocessing, so it could be generally applied to other organs or other images. It might also be extended to medical images acquired from other imaging modalities such as MRI, PET, or ultrasound.

## 5 CONCLUSION

In this paper, we proposed a CNN framework for liver segmentation. In our method, fractional differential is first used to enhance the contrast of liver and its surrounding region. CNN is then designed to produce an initial label of the liver region. Finally, maximum connectivity is applied to remove the non‐liver region. Experiment results show that our method outperforms other method in terms of several evaluation metrics. We believe that the proposed method will find its utility in more applications in the area of CT segmentation.

## CONFLICT OF INTEREST

The authors declare that there is no conflict of interest that could be perceived as prejudicing the impartiality of the research reported.

## AUTHOR CONTRIBUTION

*Conception and design*: Zhaoxuan Gong, Guodong Zhang, Wenjun Tan, Dazhe Zhao, and Cui Guo. *Development of methodology*: Zhaoxuan Gong, Wei Guo, and Guodong Zhang. *Writing, review, and/or revision of the manuscript*: Zhaoxuan Gong, Wei Guo, Guodong Zhang, Wei Zhou, and Cui Guo.

## Data Availability

The data that support the findings of this study are openly available in LiTS – Liver Tumor Segmentation Challenge (LiTS17) (URL: https://competitions.codalab.org/competitions/17094)^39^ and 3D Image Reconstruction for Comparison of Algorithm and DataBase (3Dircadb).^40^
